# Radical Cystectomy With Complete Duplication/Double Ureters

**DOI:** 10.1155/criu/6433003

**Published:** 2025-09-09

**Authors:** David S. Buchinsky, Jatan Shah, Nicolas Muruve, Jonathan Hakim

**Affiliations:** ^1^Wright State University Boonshoft School of Medicine, Dayton, Ohio, USA; ^2^Department of Pulmonology, University of Pittsburgh Medical Center, Jamestown, New York, USA; ^3^Department of Urology, Cleveland Clinic Florida, Weston, Florida, USA; ^4^Department of Urology, Dayton VA Medical Center, Dayton, Ohio, USA

## Abstract

Ureteric duplication is a variation of the urinary tract which can have significant impact in the urologic reconstructive setting. We present the incidental diagnosis and management of a 68-year-old man who was found to have a double ureter in the context of a radical cystectomy.

## 1. Introduction

Ureteric duplication is seen in < 1% of the population and is more common in females [1]. Muscle invasive bladder carcinoma has a < 15% survival rate in 2 years' time if left untreated [2]. The standard treatment of choice for muscle invasive bladder cancer is radical cystectomy and urinary diversion [3]. We present a case where an open radical cystectomy was impacted by this anatomical variation.

## 2. Case Report

**Figure 1:**
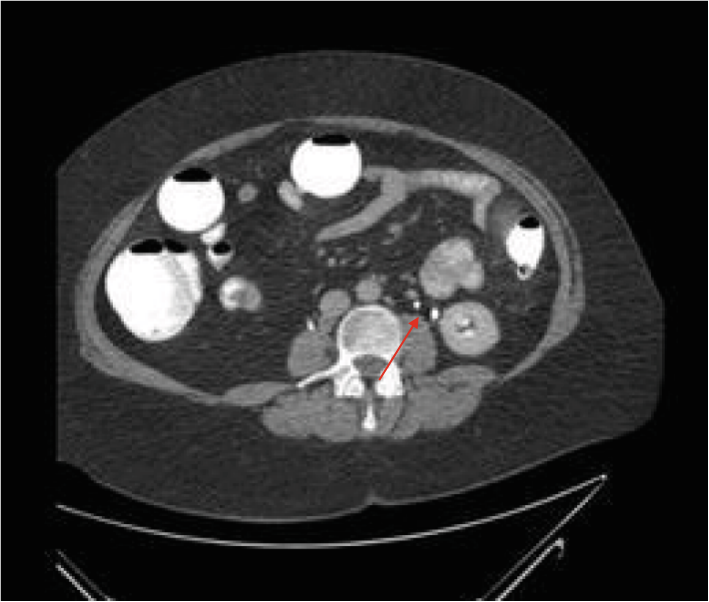
Preoperative axial view CT of the abdomen. Arrow indicates a possible left-sided double/bifid ureter in a duplex kidney around the L3 spinal level.

A 68-year-old male patient presented to the clinic with hematuria. The patient had a former smoking history of unknown duration. Computed tomography intravenous pyelogram (CT IVP) revealed a duplex left kidney with joining of the ureters (bifid ureter) at the L3 vertebral body (Figure 1). Transurethral resection of bladder tumor (TURBT) revealed muscularis propria invasive transitional cell carcinoma (TCC). He was taken to the operating room for a radical cystectomy and ileal loop diversion. Intraoperatively, the left collecting system was clinically identified as a left double ureter rather than what had been expected from prior imaging. Reconstruction was performed with left double side-to-side stented ureteroureterostomy via the Wallace technique rather than a classic Bricker technique. The final pathology was high-grade pathological stage T2b TCC of the bladder with negative margins. The patient received adjuvant chemotherapy.

## 3. Discussion

Bladder cancer is the sixth most common cancer deaths in the United States [4]. Despite previous reports from the robotic literature [5, 6], open radical cystectomy with urinary diversion remains the gold standard approach for the treatment of patients with muscle invasive bladder cancer and those with high-grade, recurrent, noninvasive tumors [3].

Renal duplication is a relatively common congenital anomaly with a reported prevalence of 0.3%–6% [7]. A duplex system refers to a kidney with two pelvicalyceal systems within a single renal parenchyma. A duplex system may contain either a single or bifid pelvis/ureter (partial duplication) or two discrete ureters (complete duplication) [8]. The majority of cases are detected during childhood, but up to 20% remain asymptomatic into adulthood [9]. In our case, the CT IVP revealed what appeared to be a left duplex system with ureters joining at approximately L3 spinal cord to become a bifid ureter. This cued the surgical team to be alert to the anatomic variation at the time of the surgery and to prepare for all inevitabilities.

Prior literature has investigated CT's ability to detect ureteral anatomic abnormalities. Eisner et al. evaluated the ability of CT in detecting ureteral duplication to determine how frequently these variations are missed in 14 patients with known ureteric duplication [10]. Their results showed that the sensitivity of axial CT with contrast material was 96%, axial CT without contrast material was 59%, and coronal CT without contrast material was 65%, respectively. In addition, Eisner et al.'s study determined negative predictive values of axial CT with contrast material (95%), axial CT without contrast material (65%), and coronal CT without contrast material (67%) [10]. It appears that duplicated ureters can be underdiagnosed on certain CT imaging protocols and that clinical urologists should be aware of this possibility.

In the robotic literature, Canda et al. described a situation where the console surgeon alertly discovered duplicated ureters that were missed by the preoperative CT [6]. Fortunately, the surgeon was able to complete the surgery robotically without complication.

In the urologic oncology reconstructive setting, the consequence of missing or failing to manage a duplex collecting system has been described. Evans et al. reported three patients in the IVP era [11]. Two of the three cases were identified as duplex kidneys on preoperative IVP, and one was not. All three developed urinomas, and the cascade of complications required additional interventions. These procedures culminated in the loss of valuable functioning renal parenchyma and included embolization of the upper pole, heminephrectomy, and total nephrectomy in the affected unit [11].

The patient in this case had a left double ureter (complete duplication) found in vivo. Therefore, just implanting the common sheath of a bifid ureter was not an option.

The Wallace technique (ureteroureterostomy) was utilized as opposed to doing separate ureteral intestinal anastomosis on the affected side (Bricker). In our case, both the frozen section and the final pathological report for the distal ureters were benign, but a theoretical risk could be exposing both poles of the kidney to potential cancer with a Wallace anastomosis.

Additionally, if there were complications with the anastomosis, such as a leak or stricture, a Wallace has only one anastomosis and is easier to detect and manage. The Wallace Y anastomosis has the lowest complication rate of any ureterointestinal anastomosis, and its stricture rate is approximately 3% [12, 13]. With the proximity of two separate anastomoses, with the classic Bricker technique, it would be difficult if not impossible to detect which one had an issue. This would necessitate revision of both which would increase the likelihood of additional problems. The disadvantage of Wallace anastomosis is that it may theoretically seed urothelial carcinoma in both upper and lower renal pole moieties of the renal unit due to its common pathway.

After performing a Wallace anastomosis, a nephroureterectomy for upper tract disease would need to be coupled with a revision of the ileal loop diversion. Luckily for our patient, both the frozen section and the final ureteral pathology were negative for upper tract TCC.

## 4. Conclusion

This case is a clarion call and warning—preoperative films should be reviewed to detect duplex kidneys and their variations. To avoid a urinoma with concomitant complications and additional procedures, duplex kidneys and their variations must be sought out and respected in the urologic oncology reconstructive setting. In our case report, Wallace anastomosis was utilized for ureter reconstruction during a radical cystectomy for T2b TCC of the bladder. Future research is warranted regarding the management of duplicated kidneys and ureters in order to minimize complications for patients.

## Data Availability

Data sharing is not applicable to this article as no datasets were generated or analyzed during the current study.

## Supporting Information

Supporting Information Additional supporting information can be found online in the Supporting Information section. Supporting figures from the literature provide additional information about the Bricker anastomosis, Wallace anastomosis, and common collecting system anomalies. Please see the supporting figures section for additional explanation.
