# Supplementary material for: Radical Cystectomy With Complete Duplication/Double Ureters
Source: Case Rep Urol. 2025 Sep 9;2025:6433003. doi: 10.1155/criu/6433003 (PMC12440662; doi:10.1155/criu/6433003)
Supplement: Supporting Information — Additional supporting information can be found online in the Supporting Information section. Supporting figures from the literature provide additional information about the Bricker anastomosis, Wallace anastomosis, and common collecting system anomalies. Please see the supporting figures section for additional explanation. [file 6433003.f1.docx]

Supplementary Figures

**Figure 1 and 2: Bricker and Wallace Anastomosis Technique**


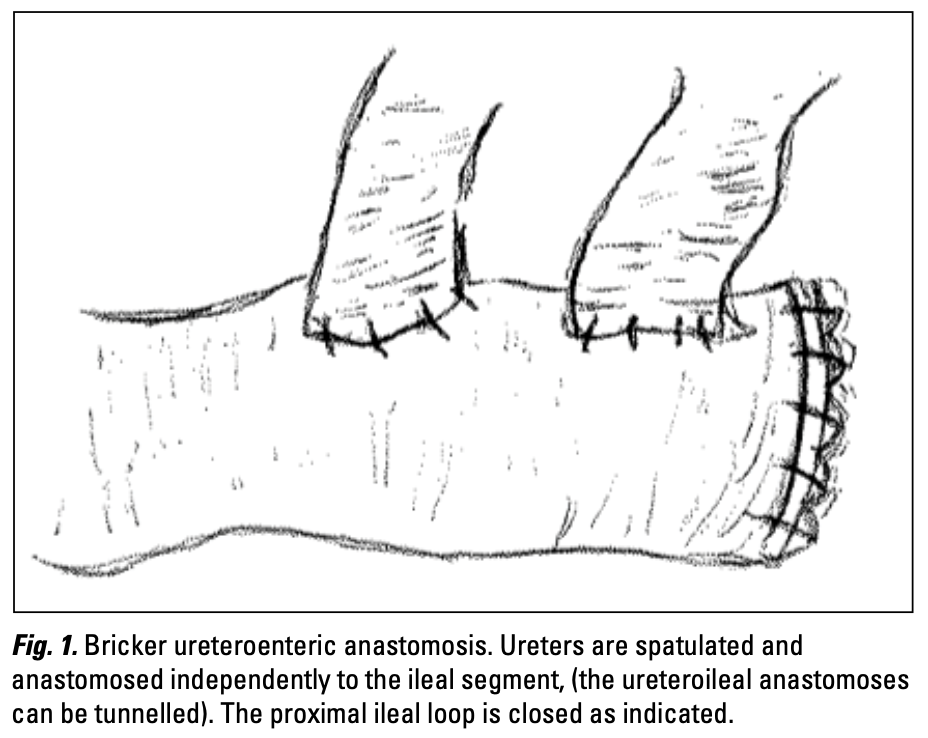


**Citation:** Davis, Niall, Burke, John, McDermott, Ted, Flynn, Robert , Manecksha, Rustom & Thornhill, John. (2015). Bricker versus Wallace anastomosis: A meta-analysis of ureteroenteric stricture rates after ileal conduit urinary diversion. Canadian Urological Association journal = Journal de l'Association des urologues du Canada. 9. E284-90. 10.5489/cuaj.2692.


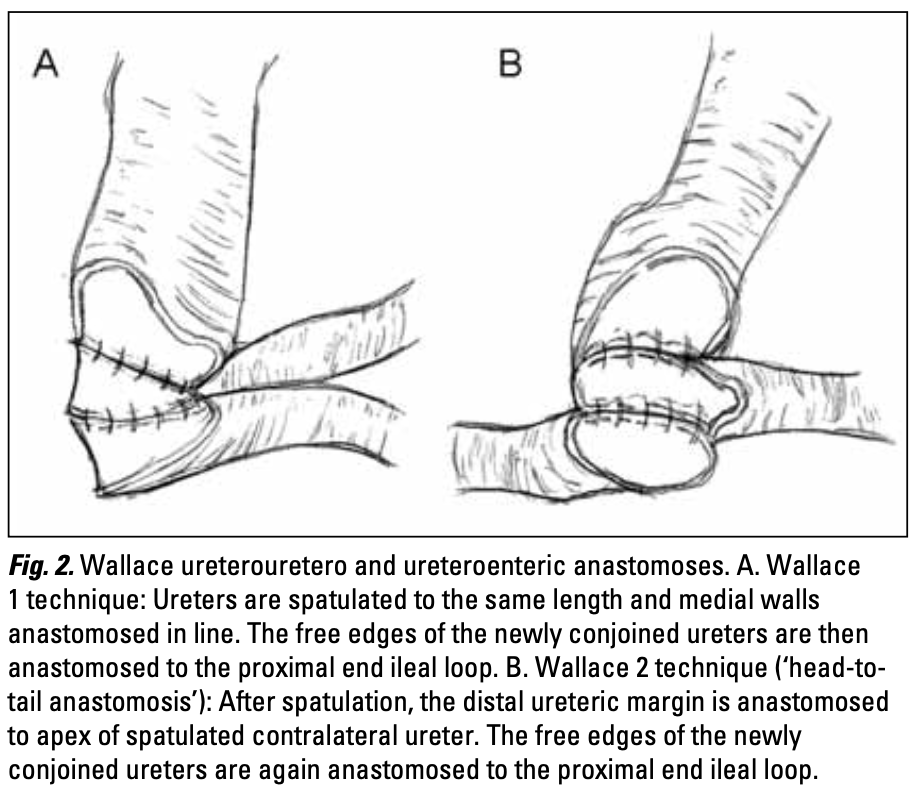


**Citation:** Davis, Niall, Burke, John, McDermott, Ted, Flynn, Robert , Manecksha, Rustom & Thornhill, John. (2015). Bricker versus Wallace anastomosis: A meta-analysis of ureteroenteric stricture rates after ileal conduit urinary diversion. Canadian Urological Association journal = Journal de l'Association des urologues du Canada. 9. E284-90. 10.5489/cuaj.2692.

**Figure 3: Common Collecting System Anomalies**


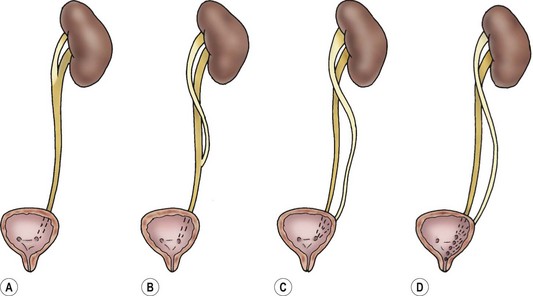


**FIGURE 54-10** Types of duplication. **(A)** Bifid pelvis. **(B)** ‘Y’ ureter. **(C)** ‘V’ ureter. **(D)** Complete duplication with various ectopic orifices.

**Citation:** Traxel EJ, Coplen DE. Ureteral obstruction and malformations. Clinical Gate. February 26, 2015. Accessed June 24, 2025. https://clinicalgate.com/ureteral-obstruction-and-malformations/.
